# Infant Formula with Added Bovine Milk Fat Globule Membrane and Modified Iron Supports Growth and Normal Iron Status at One Year of Age: A Randomized Controlled Trial

**DOI:** 10.3390/nu13124541

**Published:** 2021-12-18

**Authors:** James Hedrick, Michael Yeiser, Cheryl L. Harris, Jennifer L. Wampler, Hila Elisha London, Ashley C. Patterson, Steven S. Wu

**Affiliations:** 1 Kentucky Pediatrics, 201 South 5th Street, Bardstown, KY 40004, USA; jhedrick@bardstowncable.net; 2 Owensboro Pediatrics, 2200 E. Parrish Ave Bldg B, Suite 101, Owensboro, KY 42303, USA; myeiser@owensboropediatrics.com; 3 Medical and Scientific Affairs, Reckitt|Mead Johnson Nutrition Institute, Evansville, IN 47721, USA; cheryl.harris@rb.com (C.L.H.); jennifer.wampler@rb.com (J.L.W.); elisha.london@rb.com (H.E.L.); ashley.patterson@rb.com (A.C.P.)

**Keywords:** growth, infant feeding, infant formula, milk fat globule membrane, iron status

## Abstract

Inclusion of bovine-derived milk fat globule membrane (bMFGM) or bMFGM components in infant formulas (IFs) may support healthy brain development. This double-blind, prospective trial evaluated growth, tolerance, and iron status in infants receiving added bMFGM and modified protein, iron, and arachidonic acid (ARA) concentrations in IF. Healthy term infants were randomized to: control (marketed, routine cow’s milk-based IF/100 kcal: 2.1 g protein, 1.8 mg iron, 34 mg ARA) or INV-MFGM (investigational cow’s milk-based IF/100 kcal: 1.9 g protein, 1.2 mg iron, 25 mg ARA and whey protein-lipid concentrate, 5 g/L (source of bMFGM)). Anthropometrics, stool characteristics, fussiness, and gassiness through day 365 and blood markers of iron status at day 365 were evaluated. The primary outcome was rate of weight gain from 14–120 days of age. Of 373 infants enrolled (control: 191, INV-MFGM: 182), 275 completed the study (control: 141; INV-MFGM: 134). No group differences in growth rate (g/day) from day 14–120 or study discontinuation were detected. Few group differences in growth or parent-reported fussiness, gassiness, or stool characteristics were detected. No group differences were detected in hemoglobin, hematocrit, or incidence of anemia. In healthy term infants, bMFGM and modified protein, iron, and ARA concentrations in a cow’s milk-based IF were well-tolerated, associated with adequate growth throughout the first year of life, and supported normal iron status at one year of age.

## 1. Introduction

Milk fat globule membrane (MFGM) is a complex protein-phospholipid trilayer (2–6% of fat globule) that surrounds each fat droplet secreted into milk and is highly conserved across mammalian species [1,2,3,4]. MFGM is present in both human and bovine milk and therefore has a long history of safe use in infants and young children. However, currently marketed infant formulas typically have been designed to approximate human milk fatty acid composition using vegetable oils. Added bovine-derived MFGM (bMFGM) in infant formula may better approximate the composition of complex human milk lipids because human and bovine milk are highly homologous [5,6]. For example, total phospholipids and certain phospholipid species (including phosphatidylethanolamine, phosphatidylinositol, phosphatidylserine, and phosphatidylcholine) have been measured at comparable concentrations in bovine and human milk [7,8]. Furthermore, bMFGM ingredients (specifically Lacprodan^®^ MFGM-10, Arla Foods Ingredients P/S, Denmark) added to infant formulas have been clinically demonstrated to support normal growth and tolerance [9,10,11,12,13]. Furthermore, a growing number of studies has associated the addition of dietary bMFGM with beneficial effects on neurodevelopment [11,12,14], behavior [15], and digestive or immune health [10,16].

Protein and iron must also be considered in designing an infant formula. Expert recommendations support the use of infant formulas that have protein to fall more in line with human milk [17,18]. The ability to reformulate protein concentration closer to human milk and minimums identified by expert recommendations and regulatory provisions, while maintaining the protein quality of the infant formula, is highly dependent on protein source. Compared to human milk, total protein in infant formula has traditionally been higher to ensure sufficient protein quality to support normal growth. However, increasing evidence suggests infant formulas that have lower protein concentrations, closer to human milk, support adequate growth and potentially benefit later growth outcomes [11,19,20,21,22,23,24,25]. For iron, an important functional component of many proteins and an essential mineral for brain development, human milk content is relatively low (~0.35 mg/L) and reflected by the dietary iron requirement (0.27 mg/day) through to 6 months of age [26]. Iron fortification of infant formula is necessary to lower anemia risk in the first year of life. Most commercial formulas currently available in the United States are fortified at 12 mg iron/L (~2 mg/100 kcal, considered safe for intended use by the American Academy of Pediatrics (AAP) [27]), although the safety and nutritional suitability for lower iron, particularly for infants 0–6 months may be indicated [17,18,28,29].

Human milk also provides the long chain polyunsaturated fatty acids (LCPUFAs) docosahexaenoic acid (DHA) and arachidonic acid (ARA). Based on early human milk composition data, infant formulas that have DHA and ARA at ~0.3% and ~0.6% of total fatty acids are associated with visual and cognitive development in term infants [30,31,32,33,34]. However, a more recent comprehensive analysis of worldwide human milk composition data reported mean DHA at 0.32% and ARA at 0.47% of total fatty acids [35], and a growing body of data supports the use of infant formulas that have lower ARA content. We have previously demonstrated LCPUFA concentrations that correspond to these updated means (ARA at 25 mg/100 kcal and DHA at 17 mg/100 kcal) in infant formula support normal growth and tolerance [12,36,37,38], and red blood cell DHA has been demonstrated as equivalent to previously studied concentrations, suggesting equivalent availability for central nervous system development [38].

Regulations for growth monitoring studies have been established to ensure that new infant formulas support normal physical growth [39,40]. Consequently, the present study was designed to evaluate growth and tolerance in healthy term infants receiving formula that had an added bMFGM ingredient (5 g/L whey protein-lipid concentrate) and modified total protein (1.9 g/100 kcal, approaching the minimum identified in expert recommendations and regulatory reference ranges [17,18,39,40,41,42]), iron (1.2 mg/100 kcal), and arachidonic acid (ARA; 25 mg/100 kcal) through 365 days of age compared to a marketed, routine cow’s milk-based (2.1 g/100 kcal protein, 1.8 mg/100 kcal iron, and 34 mg/100 kcal ARA) that had no added bMFGM.

## 2. Materials and Methods

### 2.1. Study Objectives

Growth, tolerance, and adverse events were assessed from 14 to approximately 365 days of age. The rate of weight gain from 14–120 days of age was the primary variable to establish that the new formula provides adequate growth compared to a control formula. The hypothesis to be tested was that rate of weight gain using the control formula would be less than or equal to the new formula. Rejection of the hypothesis with a mean difference exceeding a clinically relevant amount (3 g/day, as outlined in American Academy of Pediatrics [AAP] guidance [43]) would indicate inadequate growth using the new formula. Failure to reject with post-study confirmation of adequate power would allow concluding that the new formula does provide adequate growth. Because the AAP recommends universal screening for iron deficiency anemia at 9–12 months of age [27], a key secondary objective was to evaluate blood markers of iron status at 365 days of age.

### 2.2. Study Design and Participants

In this multicenter, double-blind, randomized, controlled, parallel-group, prospective trial, participants were enrolled between July 2015 and November 2015 at 22 clinical sites in the United States (clinicaltrials.gov: NCT02481531). Mothers who had decided to exclusively provide infant formula were screened for study eligibility. Parents or guardians provided written informed consent prior to enrollment.

Participants were 10–14 days of age at randomization. Eligible infants were singleton births at 37–42 weeks gestational age with birth weight ≥2500 g and solely receiving formula at least 24 h prior to randomization. Exclusion criteria included: diagnosis of anemia at any time after birth or current use of iron or iron-containing supplements; history of underlying disease or congenital malformation likely to interfere with normal growth and development or participant evaluation; feeding difficulties or formula intolerance; weight at randomization <98% of birth weight; large for gestational age, born from a mother diabetic at childbirth; and immunodeficiency. Study visits corresponded to 14 (−4 days; enrollment), 30 (±3), 42 (±3), 60 (±3), 90 (±3), 120 (+5), 180 (±7), 275 (±7), and 365 (±7) days of age.

### 2.3. Randomization and Study Group Allocation

The study sponsor created a computer-generated, sex-stratified randomization schedule provided in sealed, opaque, consecutively numbered envelopes for each study site. Study formula was assigned by opening the next sequential envelope from the appropriate set at the study site. Participants were randomly assigned to receive a control (previously marketed Enfamil^®^) or investigational formula (INV-MFGM) from day 14 up to day 365 (Mead Johnson Nutrition, Evansville, IN, USA; Table 1). The source of bovine MFGM used in this study was a commercially available ingredient (whey protein-lipid concentrate added at 5 g/L; Lacprodan MFGM-10, Arla Foods Ingredients P/S, Denmark) (as reviewed [44]). Study formulas, each designated by two unique codes known only to the sponsor, were dispensed to parents at each study visit prior to study completion or withdrawal. Blinding for a participant could be broken by study sponsor personnel in the event of a medical emergency. In this study, it was not necessary to break the study code prematurely. Participants received exclusive study formula feeding through day 120. Participants who continued through day 365 were considered to complete the study even if study formula consumption discontinued or decreased to fewer than 2 feedings/day after day 180 (6 months old).

### 2.4. Study Objectives and Outcomes

The objective was to evaluate growth and tolerance in healthy, term infants. Birth anthropometric measures (body weight, length, and head circumference) were obtained from participant birth records. At all study sites, anthropometrics were recorded at days 14, 30, 42, 60, 90, 120, 180, 275, and 365 using the following standardized procedures. At each study visit, body weight was measured on a study-designated, calibrated pediatric balance (nearest g or oz); body length was measured (nearest ½ cm or ¼ in) using a recumbent pediatric stadiometer (Ellard Instrumentation, Monroe, WA, USA); and head circumference was measured (nearest ½ cm or ¼ inch) using a flexible, non-stretchable tape (infant head tape measure, Hopkins Medical Supply, Baltimore, MD, USA) provided by the study sponsor. Parents completed a baseline recall of tolerance (fussiness and gassiness) and stool characteristics (frequency and consistency) at study enrollment and 24 h recall of study formula intake, tolerance (fussiness and gassiness), and stool characteristics (frequency and consistency) at subsequent study visits. Responses were scaled for amount of gas (none = 0, slight = 1, moderate = 2, excessive = 3); fussiness (not fussy = 0, slightly = 1, moderately = 2, very = 3, extremely fussy = 4); and stool consistency (hard = 1, formed = 2, soft = 3, unformed = 4, or seedy, watery = 5). Adverse events (AEs) were coded according to specific event and the body system involved.

To assess iron status, whole blood (approximately 3 mL) was drawn via venipuncture at day 365. The following group of blood markers was selected and prioritized as follows: hemoglobin (Hb) and hematocrit (Hct); serum ferritin (SF); C-reactive protein (CRP). Per 2010 AAP guidance, a combination of blood markers is needed to accurately assess the overall iron status of a child [27]. In addition to defining iron deficiency, the AAP policy statement defines iron sufficiency as a state in which there is sufficient iron to maintain normal physiologic functions. Both Hb concentration (g/dL) and Hct, which represent the packed volume of red blood cells, are commonly used in clinical practice as determinants of anemia. Relative body iron stores are measured using SF (μg/L), but also may be elevated in a state of chronic inflammation; therefore, simultaneous measure of CRP (μg/L) is used to rule out inflammation. The Clinical Laboratory Improvement Amendment (CLIA) legal regulations are applicable to facilities that test human specimens for health assessments [45], and were passed by the United States Congress in 1988 to establish standards for “…laboratory testing to ensure the accuracy, reliability and timeliness of test results regardless of where or by whom the test was performed” [46]. In the current study, all testing facilities used to assess blood samples were CLIA-certified commercial central laboratories or institutional core laboratories using standard CLIA accepted methods of analysis (specifically, automated cell counting for Hb and Hct, immunoassay for SF, and turbidimetry for CRP). Consequently, all blood testing in the current study was performed by routine, standardized methods for clinical samples and methodology and reference ranges were comparable between testing facilities.

### 2.5. Statistical Methods

The sample size was chosen to detect a clinically relevant difference of 3 g/day in weight gain from day 14–120 (80% power; one-tailed). Assuming a standard deviation of 6.5 g/day for male and 5.5 g/day for female participants, 59 males and 43 females per study group were required to complete through day 120. Allowing for a 35% dropout rate, approximately 315 participants were targeted for enrollment. Analysis of variance (ANOVA) was used to assess growth rates in five pre-specified time intervals: from days 14 to 30, 42, 60, 90, or 120, calculated for each participant by linear regression of weight on age. Mean weight growth rates by study group and sex were compared using one-tailed tests as outlined in AAP guidelines [43].

Secondary outcomes included markers of iron status and inflammation at day 365 and anthropometrics, tolerance measures, and medically confirmed AEs through day 365. Per protocol, CRP above the laboratory reference (marker of inflammation) was analyzed (Fisher’s exact); Hb, Hct, and SF (markers of iron status) were, (1) analyzed (Kruskal–Wallis) and (2) classified relative to laboratory reference and analyzed (Cochran–Mantel-Haenszel (CMH)). For both analyses, participants with SF, but no CRP value, were included; SF was excluded when CRP was above the laboratory reference. Achieved weight, length, and head circumference; length and head circumference growth rates; formula intake; and stool frequency were analyzed by ANOVA. Stool consistency, fussiness, and gas were analyzed by CMH; AEs were analyzed by Fisher’s exact test. Post-hoc analysis compared Hb values using 2010 AAP guidelines for diagnosis and prevention of iron deficiency and iron deficiency anemia. With the exception of one-tailed tests for comparison of mean weight growth rates, all other tests were two-tailed (α = 0.05). All analyses were conducted using SAS version 9.2 (Cary, NC, USA).

## 3. Results

### 3.1. Participants

**Figure 1:**
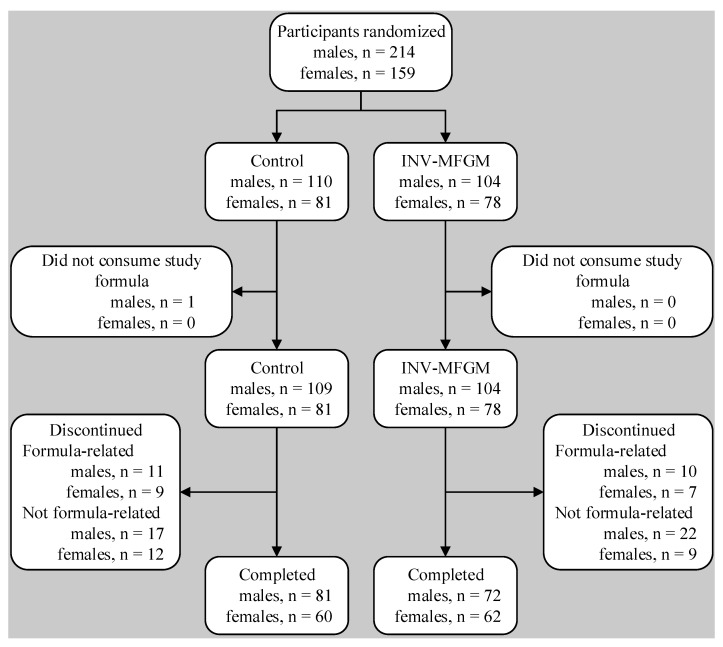
Flow of study participants.

A total of 373 participants were enrolled and randomized (control: *n* = 191; INV-MFGM: *n* = 182); 275 completed the study (control: *n* = 141; INV-MFGM: *n* = 134). Participants who were randomized but consumed no study formula (Control: *n* = 1) were excluded from analyses (Figure 1). Sex, race, and ethnic distribution and birth anthropometric measures were similar between groups (Table 2). No differences in body weight, length, or head circumference were observed by sex among groups at study enrollment.

### 3.2. Growth

**Figure 2:**
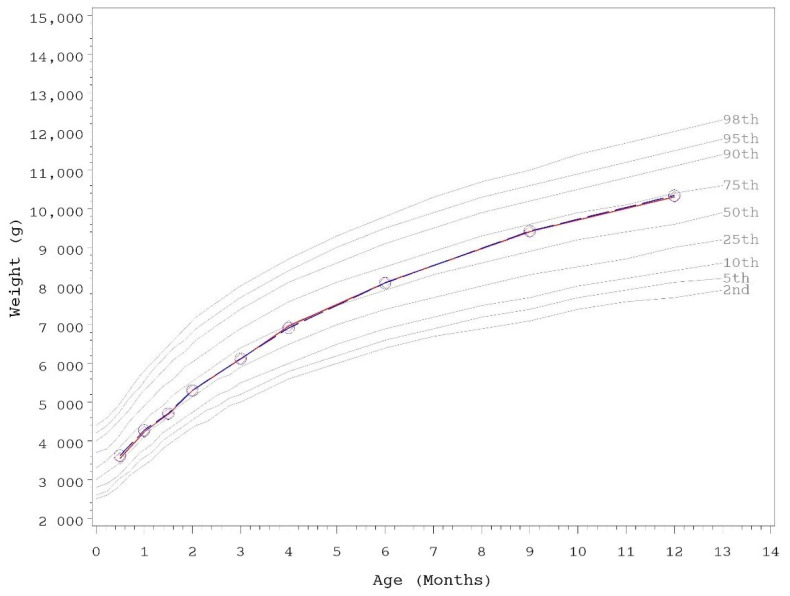
Mean achieved weight for male participants with World Health Organization (WHO) reference percentiles (2nd to 98th) through 12 months (days 14 to 365) of age. Control, red diamonds; INV-MFGM, blue circles. No statistically significant group differences by two-tailed ANOVA test.

**Figure 3:**
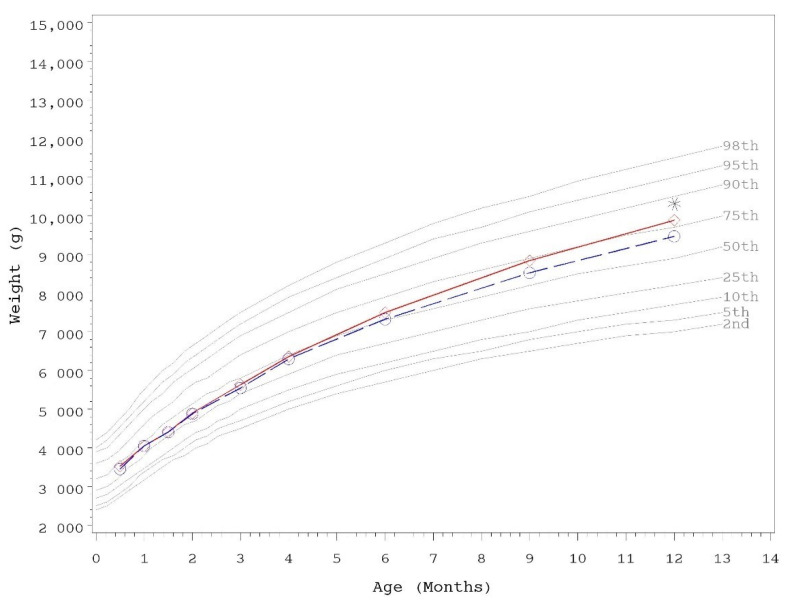
Mean achieved weight for female participants with World Health Organization (WHO) reference percentiles (2nd to 98th) through 12 months (days 14 to 365) of age. Control, red diamonds; INV-MFGM, blue circles. * Denotes significant group difference, *p* < 0.05, two-tailed ANOVA test.

Growth rates were analyzed from 14–120 days of age. As outlined in AAP guidelines, rate of weight gain is used as the most important parameter in clinical evaluation of IFs, with differences of >3 g/day over a 3–4 month period considered clinically significant [43]. No statistically significant group differences in the primary outcome, weight growth rate from day 14–120, were detected by sex (Table 3). No statistically significant group differences were detected for weight, length, or head circumference growth rates by sex for any measured range. In addition, no statistically significant differences were observed for mean achieved weight, length, or head circumference at any measured time point up to day 365 with the exception of mean achieved weight in female infants at day 365 (control, *n* = 60; 9892 ± 140, INV-MFGM, *n* = 62; 9468 ± 138; *p* = 0.034). Mean achieved weight on the WHO weight-for-age standard growth chart [47] for males (Figure 2) and females (Figure 3) in the control group remained between 25th–75th percentiles of growth through day 180 and tracked near the 75th percentile through day 365. Females in the INV-MFGM group tracked similarly through day 180 and remained between 50th–75th percentile through day 365.

### 3.3. Tolerance

There were no statistically significant group differences in parent-reported mean study formula intake (fluid oz/day) by sex at any time point assessed or in mean duration (days) of study formula intake (Table 4). After day 180, mean reported study formula intake began to generally decline for all participants as expected, as parents and caregivers likely begin to offer complementary foods to infants at approximately 4–6 months of age. Parent-reported gassiness and fussiness were similar among groups at all time points (data not shown). Using 24 h recall, the amount of gas most commonly reported was “slight amount” or “moderate amount” up to 180 days of age, and “none at all” or “slight amount” by days 275 and 365. Fussiness was most often characterized as “slightly fussy” or “not fussy”. No significant group differences in stool frequency or consistency were detected at any time point assessed (Table 5), with the exception of stool consistency at day 90. By category, the primary differences at this time point were fewer infants with soft and more with unformed or seedy stool consistency in the INV-MFGM compared to the control group.

In the overall study population (all participants up to day 365), no statistically significant group differences were detected for study formula discontinuation, either related (control: 20, 11%; INV-MFGM: 17, 9%) or not related to study formula (control: 57, 30%; INV-MFGM: 56, 31%). For formula-related discontinuation, formula intolerance determined by the study investigator was the most common reason (control: 20; INV-MFGM: 15); fussiness (control: 9; INV-MFGM: 7) and gas (control: 8; INV-MFGM: 5) were the most common symptoms. Parental decision was the most common reason for discontinuation not related to study formula (control: 22; INV-MFGM: 24). No group difference was detected in the number of participants for whom at least one medically-confirmed AE was reported (control: 176, 93%; INV-MFGM: 166; 91%). No statistically significant group differences were detected in the incidence of medically confirmed AEs by system: body as a whole; cardiovascular; endocrine; eyes, ear, nose and throat; gastrointestinal (GI); metabolic and nutrition; musculoskeletal; nervous system; skin; respiratory; and urogenital. Within the skin system, group incidence of medically-confirmed eczema was similar (control: 34, 18%; INV-MFGM: 32, 18%; *p* = 1.000). Within the eyes, ears, nose, and throat system, nasal/tear duct obstruction incidence was significantly different (control: 18, 9%; INV-MFGM: 6, 3%; *p* = 0.019). Within the GI system, gas incidence was significantly lower for INV-MFGM (9, 5%) versus control (24, 13%; *p* = 0.010). Within the “feeding problem” category, AE incidence was low but statistically significant (control: 0, 0%; INV-MFGM: 7, 4%; *p* = 0.006); assorted AEs included feeding difficulty/intolerance, including that associated with beginning complementary foods (mild, 5) and newborn feeding problems (mild, 1; moderate, 1). No group differences were detected in the incidence of AEs associated with allergic manifestations or infection. For 30 participants (control: 17, 9%; INV-MFGM: 13, 7%) who experienced serious adverse events, all were assessed as unrelated to study formulas by study physicians, with the exception of one infant (INV-MFGM) considered intolerant to study formula (later diagnosed with esophageal reflux) and one infant (INV-MFGM) diagnosed with cow milk protein allergy after study enrollment.

### 3.4. Iron Status

Blood markers of iron status evaluated at day 365 (control: 141; INV-MFGM: 127) were a key secondary outcome (Table 6). No significant differences were detected between study formula groups in actual Hb (g/dL) or Hct (%) or relative to laboratory references, and the majority of participants fell within reference ranges for Hb (control: 88%; INV-MFGM: 91%) and Hct (control: 87%; INV-MFGM: 87%). SF was significantly higher (*p* = 0.048) for control versus the INV-MFGM group. However, no significant group differences were detected relative to laboratory references and the majority of participants fell within SF reference ranges (control: 84%; INV-MFGM: 90%). Few participants were diagnosed with anemia during the study period (recorded as individual AEs) (control: 5, 3%; INV-MFGM: 1, 1%). By post-hoc analysis, no significant group difference was detected using the 2010 AAP Hb ≥ 11.0 g/dL cutoff for anemia evaluation (control: 91%; INV-MFGM: 87%).

No significant group difference in C-reactive protein relative to the laboratory reference was detected with most participants falling below the upper reference ranges (control: 95%; INV-MFGM: 92%) (Table 7).

### 3.5. C-reactive Protein

No significant group difference in C-reactive protein relative to the laboratory reference was detected with most participants falling below the upper reference ranges (control: 95%; INV-MFGM: 92%) (Table 7).

## 4. Discussion

In healthy infants, the addition of a bMFGM ingredient and iron at 1.2 mg/100 kcal, protein at 1.9 g/100 kcal, and ARA at 25 mg/100 kcal in a routine cow’s milk-based infant formula was well-tolerated and associated with adequate growth throughout the first year of life and supported normal iron status at one year of age. Safety and potential benefits of adding a bMFGM ingredient (in particular MFGM-10) or its components in infant formula [9,10,11,13,14,48] or complementary food for infants and young children [16] have been previously demonstrated. Comparable modifications in concentrations of protein [49], iron [50], and ARA [38] have also been tested separately and shown to support adequate growth and be well tolerated. We also recently demonstrated an accelerated neurodevelopmental profile by 12 months of age in infants receiving the same added bMFGM ingredient and bovine lactoferrin in infant formula at concentrations similar to human milk through 12 months of age [12]. The current study is the first large pediatric nutrition trial designed to evaluate an added bMFGM ingredient (MFGM-10) in addition to modified iron, protein, and ARA concentrations.

In the current study, acceptance and tolerance of study formulas were good. No differences in overall study discontinuation or study discontinuation due to study formula were detected. No significant group differences were detected in fussiness, gassiness, or mean stool frequency at any measured time point. Few group differences from day 30 to 365 were reported for stool consistency. In one previous clinical study of infants receiving formula with added bMFGM, post-hoc analysis suggested that eczema incidence was low, but increased in the group receiving added bMFGM in formula [9]. In the present trial, there were no significant group differences in eczema incidence or the overall incidence of AEs within the skin system. Previous studies using the same source of bMFGM (MFGM-10) in formula demonstrated no association between bMFGM and increased risk of eczema through 6 or 18 months of age [11,12]. Overall, the present study adds to the growing body of evidence that the addition of MFGM-10 to formulas for infants is well-tolerated.

In the current study, adequate growth exhibited by infants receiving the investigational formula demonstrates nutritional suitability of a formula protein blend at a protein concentration (1.9 g/100 kcal) that approaches the minimum identified in expert recommendations and regulatory reference ranges [17,18,39,40,41,42], and includes added MFGM-10. Previous studies have demonstrated formula protein blends with added bMFGM and similar total protein concentration (~1.9–2.0 g/100 kcal) support adequate growth in infants [9,11]. In infants receiving MFGM-10, a different lipid-rich bMFGM ingredient, or standard formula (no added bMFGM) with similar total protein, no significant group differences in weight gain or growth were demonstrated and weight Z-scores indicated normal growth [9]. Growth was also similar in infants receiving formulas that had added MFGM-10 and adapted energy (60 kcal/100 mL) vs. a control with no added bMFGM and higher energy (66 kcal/100 mL), although mean formula intake was higher for the former group [11]. Demonstration of adequate growth in the present study is evidence of adequate protein quality of formula that has a blend of bovine-derived protein (including added MFGM-10) at 1.9 g/100 kcal.

Recent expert recommendations have supported the conclusion that lower iron content is sufficient to support healthy growth and iron fortification at 1.2 mg/100 kcal, which complies with regulatory provisions [39,40,41,51] and aligns with expert recommendations applicable to formulas designed for infants 0–12 months of age [18,28,29,52,53,54]. In the current study, no significant group differences in Hb or Hct at day 365 were detected, and the majority of participants fell within laboratory reference ranges. Consistent with higher dietary iron intake, serum ferritin was significantly higher in participants receiving the control versus investigational formula, but fell within or above laboratory reference ranges in all but six study participants: three (control) had been considered iron deficient and received medically prescribed iron supplements; three (control, 1; INV-MFGM, 2) were classified iron deficient based on laboratory references but iron sufficient based on 2010 AAP cutoffs. In addition, there were no group differences in physiologically relevant outcomes (including anemia, iron deficiency).

A key strength of this study included the randomized, double-blind, controlled design. In addition, because no single measure can characterize iron status, a combination of blood markers recommended by the AAP and commonly used in clinical practice was assessed. In addition, the day 365 study timepoint when iron status was evaluated corresponded with the AAP recommendation for universal screening for iron deficiency at 12 months of age [27]. Although the current study did not include a reference group of infants exclusively receiving human milk for comparison, in accordance with AAP guidance [43], the investigational formula was compared to a previously marketed formula demonstrated to support adequate growth in infants. In addition, WHO reference standards, which are representative of typical growth in breastfed infants, were used to plot current growth data. Finally, although study formula intake was recorded in the current study through 365 days of age, participants were exclusively receiving study feeding through 120 days of age only. After this age, infants may begin complementary feeding, which will also impact growth and iron status. Collecting complementary feeding in addition to study formula intake could provide a more complete dietary recall throughout the first year of life and may be warranted in future infant nutrition studies.

## 5. Conclusions

Overall, intact cow’s milk protein infant formula with the addition of a bMFGM ingredient and iron at 1.2 mg/100 kcal, protein at 1.9 g/100 kcal, and ARA at 25 mg/100 kcal was well-tolerated and associated with age-appropriate growth throughout the first year of life. Blood markers and physiological outcomes associated with iron status were within normal ranges in infants receiving added bMFGM in formula and modified protein, iron, and ARA. Consequently, this study demonstrated that added bMFGM and modified protein, iron, and ARA concentration in a routine cow’s milk infant formula were safe, well-tolerated, and associated with adequate growth throughout the first year of life and supported normal iron status at one year of age.

## Figures and Tables

**Table 1:** Nutrient composition per 100 kcal (20 Calories/fl oz).

Nutrient	Study Formula (Target Values)	
	Control	INV-MFGM
Total Protein, g ^†^	2.1	1.9
Total Fat, g ^‡^	5.3	5.3
ARA, mg ^‡^	34	25
DHA, mg ^‡^	17	17
Total Carbohydrate, g ^§^	11.2	11.4
Vitamin A, IU	300	300
Vitamin D, IU	60	60
Vitamin E, IU	2	2
Vitamin K, mcg	9	9
Thiamin, mcg	80	80
Riboflavin, mcg	140	140
Vitamin B6, mcg	60	60
Vitamin B12, mcg	0.3	0.3
Niacin, mcg	1000	1000
Folic Acid, mcg	16	16
Pantothenic Acid, mcg	500	500
Biotin, mcg	3	3
Vitamin C, mg	12	12
Choline, mg	24	24
Inositol, mg	6	6
Calcium, mg	78	78
Phosphorus, mg	43	43
Magnesium, mg	8	8
Iron, mg	1.8	1.2
Zinc, mg	1	1
Manganese, mcg	15	15
Copper, mcg	75	75
Iodine, mcg	15	15
Selenium, mcg	2.8	2.8
Sodium, mg	27	27
Potassium, mg	108	108
Chloride, mg	63	63

^†^ Sources of protein for control: skim milk and whey protein concentrate (WPC); and for INV-MFGM: skim milk, WPC, and whey protein-lipid concentrate (5 g/L, source of bMFGM; Lacprodan^®^ MFGM-10, Arla Foods Ingredients P/S, Denmark). ^‡^ Sources of fat: base blend of palm olein, soybean, coconut, and high oleic sunflower oils; fungal-derived single cell oil (source of ARA); algal-derived single cell oil (source of DHA). ^§^ Available carbohydrate: control = 10.6 g; INV-MFGM = 10.8 g; prebiotic oligosaccharides = 0.6 g (source: prebiotic blend of polydextrose (PDX, Litesse^®^ Two Polydextrose; Danisco) and galactooligosaccharides (GOS, [Vivinal^®^ GOS Galactooligosaccharide; Friesland Foods Domo]. 1:1 ratio, 4 g/L)).

**Table 2:** Infant characteristics at birth and study entry.

Infant Characteristic	Study Group		*p*
	Control	INV-MFGM	
Sex, *n* (%) ^a^			1.000
Female	81 (43)	78 (43)	
Male	109 (57)	104 (57)	
Race, *n* (%) ^a^			0.371
White	156 (83)	142 (78)	
Black	20 (11)	20 (11)	
Other	13 (7)	20 (11)	
Ethnicity, *n* (%) ^a^			1.000
Hispanic	13 (7)	12 (7)	
Not Hispanic	177 (93)	170 (93)	
Birth anthropometrics ^b^	Study Group, *n*	mean ± SE	*p*
Weight (g)	Control, 190	3374.3 ± 30.3	0.655
	INV-MFGM, 182	3393.5 ± 31.0	
Length (cm)	Control, 188	50.9 ± 0.2	0.676
	INV-MFGM, 182	50.8 ± 0.2	
Head circumference (cm)	Control, 185	34.3 ± 0.1	0.884
	INV-MFGM, 181	34.3 ± 0.1	
Anthropometrics at Study Entry ^b^	Study Group, *n*	mean ± SE	*p*
males			
Weight (g)	Control, 109	3550.6 ± 38.5	0.187
	MFGM-10, 104	3623.6 ± 39.4	
Length (cm)	Control, 109	51.7 ± 0.2	0.595
	MFGM-10, 104	51.9 ± 0.2	
Head circumference (cm)	Control, 109	36.1 ± 0.1	0.947
	MFGM-10, 104	36.1 ± 0.1	
females			
Weight (g)	Control, 81	3526.4 ± 43.2	0.233
	INV-MFGM, 77	3452.2 ± 44.3	
Length (cm)	Control, 81	51.3 ± 0.2	0.729
	INV-MFGM, 78	51.2 ± 0.2	
Head circumference (cm)	Control, 81	35.6 ± 0.1	0.625
	INV-MFGM, 78	35.5 ± 0.1	

^a^ Fisher’s exact test. ^b^ Two-tailed ANOVA test.

**Table 3:** Weight, length, and head circumference growth rates from 14 days to 30, 42, 60, 90, and 120 days of age ^a^.

Day	Study	Weight ^b^			Length ^c^			Head Circumference ^c^		
	Group	*n*	g/day	*p*	*n*	cm/day	*p*	*n*	cm/day	*p*
males										
30	Control	104	40.0 ± 1.1	0.115	103	0.13 ± 0.007	0.644	103	0.09 ± 0.003	0.955
	INV-MFGM	101	38.2 ± 1.1		101	0.13 ± 0.007		101	0.09 ± 0.003	
42	Control	98	39.0 ± 0.9	0.137	98	0.12 ± 0.005	0.321	98	0.09 ± 0.002	0.55
	INV-MFGM	96	37.5 ± 0.9		96	0.13 ± 0.005		96	0.08 ± 0.002	
60	Control	96	36.5 ± 0.9	0.191	96	0.12 ± 0.003	0.869	96	0.07 ± 0.002	0.965
	INV-MFGM	92	35.4 ± 0.9		92	0.12 ± 0.003		92	0.07 ± 0.002	
90	Control	91	33.1 ± 0.8	0.245	91	0.12 ± 0.002	0.268	91	0.06 ± 0.001	0.602
	INV-MFGM	85	32.3 ± 0.8		84	0.11 ± 0.002		84	0.06 ± 0.001	
120	Control	86	30.5 ± 0.7	0.271	86	0.11 ± 0.002	0.53	86	0.05 ± 0.001	0.596
	INV-MFGM	81	29.9 ± 0.7		81	0.11 ± 0.002		81	0.06 ± 0.001	
females										
30	Control	70	31.7 ± 1.3	0.723	70	0.12 ± 0.008	0.699	70	0.08 ± 0.004	0.432
	INV-MFGM	71	32.7 ± 1.3		72	0.12 ± 0.008		72	0.08 ± 0.004	
42	Control	68	31.0 ± 1.1	0.551	68	0.12 ± 0.005	0.675	68	0.08 ± 0.003	0.215
	INV-MFGM	69	31.2 ± 1.1		70	0.12 ± 0.005		70	0.07 ± 0.003	
60	Control	66	29.2 ± 1.0	0.452	66	0.11 ± 0.003	0.868	66	0.06 ± 0.002	0.334
	INV-MFGM	66	29.0 ± 1.0		67	0.11 ± 0.003		67	0.07 ± 0.002	
90	Control	64	27.1 ± 0.8	0.309	64	0.10 ± 0.002	0.534	64	0.06 ± 0.001	0.863
	INV-MFGM	65	26.5 ± 0.8		66	0.11 ± 0.002		66	0.06 ± 0.001	
120	Control	62	25.5 ± 0.7	0.343	61	0.10 ± 0.002	0.834	61	0.05 ± 0.001	0.813
	INV-MFGM	64	25.1 ± 0.7		65	0.10 ± 0.002		65	0.05 ± 0.001	

^a^ Mean ± standard error (SE). ^b^ One-tailed ANOVA test. ^c^ Two-tailed ANOVA test.

**Table 4:** Study formula intake (fluid oz/day) at days 30, 42, 60, 90, 120, 180, 275, and 365 *.

Age (Days)			Male			Female			
	Study Group	*n*	Mean	(s.e.)	*p*	*n*	Mean	(s.e.)	*p*
30	Control	97	30.1	(0.9)	0.331	70	27.5	(1.0)	0.777
	INV-MFGM	97	28.8	(0.9)		70	27.9	(1.0)	
42	Control	96	32.2	(0.9)	0.999	68	29.7	(0.8)	0.431
	INV-MFGM	92	32.2	(0.9)		68	28.8	(0.8)	
60	Control	92	34.8	(1.0)	0.951	63	31.8	(0.8)	0.131
	INV-MFGM	90	34.9	(1.0)		67	30.1	(0.8)	
90	Control	87	38.4	(1.1)	0.849	61	34.5	(1.1)	0.647
	INV-MFGM	83	38.7	(1.1)		66	33.8	(1.0)	
120	Control	85	40.6	(1.3)	0.345	58	36.4	(1.0)	0.348
	INV-MFGM	79	38.9	(1.3)		65	35.1	(1.0)	
180	Control	84	39.2	(1.2)	0.932	61	38.2	(1.2)	0.269
	INV-MFGM	74	39.1	(1.3)		63	36.4	(1.2)	
275	Control	83	36.8	(1.1)	0.856	59	33.6	(1.5)	0.167
	INV-MFGM	74	37.1	(1.2)		62	36.5	(1.4)	
365	Control	76	30.0	(1.2)	0.533	55	31.2	(1.6)	0.487
	INV-MFGM	67	28.9	(1.3)		55	29.6	(1.6)	

* Mean ± standard error (SE); for reference, 1 oz = 29.57 milliliters; analyzed by two-tailed ANOVA test.

**Table 5:** Stool characteristics at days 14, 30, 42, 60, 90, 120, 180, 275, and 365.

		Stool Frequency ^a^			Stool Consistency, *n* (%) ^b^					
Day	Group	*n*	Mean ± SE	*p*	Hard	Formed	Soft	Unformed or Seedy	Watery	*p*
14	Control	190	3.3 ± 0.2	0.182	2 (1)	5 (3)	86 (46)	93 (49)	2 (1)	0.249
	INV-MFGM	182	3.7 ± 0.2		1 (1)	4 (2)	74 (41)	97 (54)	3 (2)	
30	Control	166	2.9 ± 0.1	0.416	0 (0)	1 (1)	67 (41)	88 (54)	8 (5)	0.926
	INV-MFGM	167	2.7 ± 0.1		1 (1)	1 (1)	62 (38)	93 (57)	7 (4)	
42	Control	162	2.4 ± 0.1	0.303	1 (1)	0 (0)	68 (43)	84 (53)	7 (4)	0.308
	INV-MFGM	159	2.2 ± 0.1		2 (1)	4 (3)	66 (44)	68 (46)	9 (6)	
60	Control	154	2.1 ± 0.1	0.986	0 (0)	3 (2)	80 (53)	62 (41)	7 (5)	0.221
	INV-MFGM	153	2.1 ± 0.1		0 (0)	1 (1)	71 (47)	70 (47)	8 (5)	
90	Control	146	2.3 ± 0.1	0.804	0 (0)	4 (3)	77 (55)	56 (40)	3 (2)	0.004 *
	INV-MFGM	147	2.2 ± 0.1		0 (0)	1 (1)	63 (44)	69 (48)	11 (8)	
120	Control	139	2.2 ± 0.1	0.843	1 (1)	4 (3)	81 (57)	50 (35)	5 (4)	0.094
	INV-MFGM	143	2.1 ± 0.1		2 (1)	0 (0)	71 (50)	61 (43)	8 (6)	
180	Control	144	2.5 ± 0.1	0.104	1 (1)	8 (6)	105 (72)	30 (21)	1 (1)	0.053
	INV-MFGM	136	2.2 ± 0.1		1 (1)	6 (5)	86 (65)	31 (23)	8 (6)	
275	Control	142	2.3 ± 0.1	0.997	1 (1)	24 (17)	95 (68)	16 (11)	4 (3)	0.991
	INV-MFGM	136	2.3 ± 0.1		0 (0)	23 (17)	91 (69)	15 (11)	3 (2)	
365	Control	131	2.1 ± 0.1	0.333	5 (4)	32 (25)	80 (62)	6 (5)	6 (5)	0.411
	INV-MFGM	120	2.3 ± 0.1		3 (3)	25 (21)	74 (62)	16 (13)	1 (1)	

^a^ Two-tailed ANOVA test. ^b^ Cochran–Mantel–Haenszel row mean score test. * Significantly different (*p <* 0.05).

**Table 6:** Hemoglobin, hematocrit, and serum ferritin at day 365.

		Concentration ^a^				Relative to Laboratory References, *n* (%) ^b^				Relative to 2010 AAP Guidelines ^c^, *n* (%)		
	Study Group	*n*	Median	IQR	*p*	Below	Within	Above	*p*	Hb < 11.0 g/dL ^d^	Hb ≥ 11.0 g/dL	*p*
Hemoglobin, g/dL	Control	141	12.1	11.5–12.6	0.275	14 (10)	124 (88)	3 (2)	0.703	13 (9)	128 (91)	0.433
	INV-MFGM	127	12.0	11.3–12.4		10 (8)	115 (91)	2 (2)		16 (13)	111 (87)	
Hematocrit, %	Control	141	35.9	34.2–37.7	0.262	15 (11)	122 (87)	4 (3)	0.389			
	INV-MFGM	126	35.5	33.9–37.1		11 (9)	109 (87)	6 (5)				
Serum Ferritin, μg/L	Control	129	50.0	32.3–65.0	0.048 *	4 (3)	109 (84)	16 (12)	0.472			
	INV-MFGM	114	42.0	28.8–59.0		2 (2)	104 (90)	9 (8)				

^a^ Kruskal–Wallis test. ^b^ Cochran–Mantel–Haenszel row mean score test. ^c^ Using American Academy of Pediatrics (AAP) cutoffs in practice, an initial measure of Hb < 11.0 g/dL (recommended cutoff) would prompt further evaluation for iron deficiency anemia by measuring SF (AAP suggested cutoff < 10.0 μg/L) and CRP to characterize iron status. Analyzed by Fisher’s exact test. ^d^ For 29 participants (control: 13, 9%; INV-MFGM: 16, 13%) categorized as hemoglobin (Hb) < 11.0 g/dL, 21 (control: 8: INV-MFGM: 13) were classified as having serum ferritin (SF) ≥10.0 μg/L (values lower than the 2010 AAP suggested cutoff would denote depletion of iron stores); 3 (control) had received medically prescribed iron supplements prior to the blood draw at day 365 and were therefore classified as iron deficient by the study investigator. Iron status could not be assessed in five participants (control: 2; INV-MFGM: 3) because SF was not obtained or was invalid due to elevated C-reactive protein (CRP). * Statistically significant, *p* < 0.05.

**Table 7:** C-reactive protein relative to laboratory references at day 365.

C-reactive Protein Relative to Laboratory References ^a^	Study Group, *n* (%)		*p*
	Control	INV-MFGM	
Not Above	128 (95)	113 (92)	0.342
Above	7 (5)	10 (8)	

^a^ Fisher’s exact test.

## Data Availability

The authors and study sponsor encourage and support the responsible and ethical sharing of data from clinical trials. De-identified participant data from the final research dataset used in the published manuscript may only be shared under the terms of a Data Use Agreement. Requests may be directed to: steven.wu2@rb.com.

## Abbreviations

American Academy of Pediatrics, AAP; arachidonic acid, ARA; C-reactive protein, CRP; docosahexaenoic acid, DHA; hematocrit, Hct; hemoglobin, Hb; long chain polyunsaturated fatty acids, LCPUFAs; milk fat globule membrane, MFGM; serum ferritin, SF; World Health Organization, WHO.
